# Association of hair iron levels with creativity and psychological variables related to creativity

**DOI:** 10.3389/fnhum.2013.00875

**Published:** 2013-12-18

**Authors:** Hikaru Takeuchi, Yasuyuki Taki, Atsushi Sekiguchi, Rui Nouchi, Yuka Kotozaki, Seishu Nakagawa, Carlos M. Miyauchi, Kunio Iizuka, Ryoichi Yokoyama, Takamitsu Shinada, Yuki Yamamoto, Sugiko Hanawa, Tsuyoshi Araki, Hiroshi Hashizume, Keiko Kunitoki, Yuko Sassa, Ryuta Kawashima

**Affiliations:** ^1^Division of Developmental Cognitive Neuroscience, Institute of Development, Aging and Cancer, Tohoku UniversitySendai, Japan; ^2^Division of Medical Neuroimaging Analysis, Department of Community Medical Supports, Tohoku Medical Megabank Organization, Tohoku UniversitySendai, Japan; ^3^Department of Radiology and Nuclear Medicine, Institute of Development, Aging and Cancer, Tohoku UniversitySendai, Japan; ^4^Department of Functional Brain Imaging, Institute of Development, Aging and Cancer, Tohoku UniversitySendai, Japan; ^5^Human and Social Response Research Division, International Research Institute of Disaster Science, Tohoku UniversitySendai, Japan; ^6^Smart Ageing International Research Center, Institute of Development, Aging and Cancer, Tohoku UniversitySendai, Japan; ^7^Graduate Schools for Law and Politics, The University of TokyoBunkyo, Tokyo, Japan; ^8^Japan Society for the Promotion of ScienceTokyo, Japan; ^9^Faculty of Medicine, Tohoku UniversitySendai, Japan

**Keywords:** hair minerals, iron, creativity, dopamine, novelty seeking, motivation, extraversion, physical activity

## Abstract

Creativity generally involves the conception of original and valuable ideas. Previous studies have suggested an association between creativity and the dopaminergic system, and that physical activity facilitates creativity. Iron plays a key role in the dopaminergic system and physical activity. Here, we newly investigated the associations between hair iron levels and creativity, dopamine-related traits and states [novelty seeking, extraversion, and vigor (motivational state)], as well as the physical activity level. In the present study, we addressed this issue by performing a hair mineral analysis to determine iron levels and a behavioral creativity test of divergent thinking and related psychological measures among young adults (254 men, 88 women; mean age 20.79 ± 2.03 years). Iron levels did not show any significant association with creativity but displayed significant positive associations with novelty seeking, extraversion, and physical activity level. These results may be partly congruent with the notion that iron plays a key role in the dopaminergic system and imply that iron is important for traits and physical activity, which facilitate creativity. Future interventional or longitudinal studies are warranted to identify any causal effects.

## Introduction

The broadly accepted standard definition of creativity is the ability to produce work that is both novel and useful within a certain social context (Stein, 1953; Runco and Jaeger, 2012). Creative cognition has been essential to the development of human civilization and plays a crucial role in cultural life. Divergent thinking (DT) involves information retrieval and calls for a varied responses; it has been proposed as a key aspect of creative cognition (Guilford, 1967), and its strong predictive validity has been identified through a meta-analysis (Kim, 2008).

Previous psychological, neuropsychological and functional imaging studies have indicated that the dopaminergic system contributes to creative cognition. These findings can be classified as (1) associations between creativity and schizotypy (Kline and Cooper, 1986; Eysenck and Furnham, 1993; Cooper, 1998; O'reilly et al., 2001), which is associated with dopamine-related genes (Ettinger et al., 2006) and overactivity of subcortical dopaminergic systems (Kirrane and Siever, 2000), (2) associations between creativity and novelty seeking (Chavez-Eakle et al., 2006), which has been associated with dopaminergic functions various studies (Suhara et al., 2001; Schinka et al., 2002; Kaasinen et al., 2004; Tomer and Aharon-Peretz, 2004; Bódi et al., 2009), (3) associations between creativity and motivation that are not caused by external incentives (Prabhu et al., 2008), which has been associated with dopaminergic functions (Kaplan and Oudeyer, 2007), (4) associations between creativity and extraversion (King et al., 1996), which has been associated with dopaminergic functions (Ashby and Isen, 1999; Depue and Collins, 1999), and (5) dopamine's antagonist's effects to suppress creativity (Flaherty, 2005) as well as dopamine's effects to suppress latent inhibition (a behavioral index of the ability to habituate to sensations) (Ellenbroek et al., 1996; Swerdlow et al., 2003), while reduced latent inhibition is associated with creativity among intelligent subjects (Carson et al., 2003). Furthermore, recent neuroimaging studies have shown an association between creativity and dopamine receptor binding potential, as well as with brain structures in the dopaminergic system (De Manzano et al., 2010; Takeuchi et al., 2010a). These reports are consistent with the findings of integrative reviews of creative cognition, which suggest that the dopaminergic system is associated with creative cognition (Heilman et al., 2003; Flaherty, 2005).

Certain mineral levels in the body and dietary mineral intake have been associated with dopaminergic functions. Some metals, such as iron and copper, have long been suggested to play essential roles in dopaminergic systems, and the amount of these metals in the brain is assumed to be associated with dopaminergic functions (Pfeiffer and Mailloux, 1987; Ortega et al., 2007). In particular, iron deficiency in humans results in poorer performance, and malfunctioning of the dopaminergic system has been suggested as a mechanism underlying this poor performance (McCann and Ames, 2007). In detail, iron is required by enzymes involved in the synthesis of dopamine (tyrosine hydroxylase) (McCann and Ames, 2007), and a reduction in the density and affinity of dopamine D2 receptors is associated with iron restrictions in rodents (Yehuda and Youdim, 1989; Beard et al., 1993; Youdim and Yehuda, 2000). These changes are thought to be responsible for the effects of iron restriction on motor, cognitive, or behavioral performance (Yehuda and Youdim, 1989; Beard, 2001; Beard and Connor, 2003). Whether cognitive or behavioral deficits result from iron deficiency without anemia is an open question, but the available evidence suggests that they do (for a review, see McCann and Ames, 2007). For example, there is some evidence that iron repletion can facilitate cognitive performance even in the absence of anemia (Sandstead, 2000) and that subjects with iron deficiency without anemia show deficits in the performance of cognitive tasks and abnormal electroencephalography activity patterns (Otero et al., 1999, 2004; for a review, see McCann and Ames, 2007). Finally, higher levels of negative affects, lower levels of attention to people and objects, and lower activity levels were observed in infants with iron deficiency anemia as well as in infants with lower hemoglobin levels and serum iron levels (Lozoff et al., 1996, 1998; Wachs et al., 2005). In addition, iron supplementation in infants increases the positive affects and attention toward people in their environment (Lozoff et al., 2003).

However, despite these studies and vast amount of research investigating the hair mineral levels and cognitive functions, the associations between body iron levels and creativity have not yet been investigated. We hypothesized that creativity is positively correlated with iron levels in the body, considering the role of iron in creativity and the association of dopaminergic function with creativity described above. Further, iron is critical to the formation of hemoglobin and red blood cells, which transport (Hata, 1982) oxygen to the body and without which efficient physical activity is impossible, the higher physical activity level has been associated with higher creativity (Cavallera et al., 2011). In addition, robust evidence is available that participation in aerobic exercise and sports facilitates creativity (Tuckman and Hinkle, 1986; Welsh and Labbé, 1994; Zachopoulou et al., 2006). Therefore, higher iron levels may be associated with the facilitation of creativity through the increased physical activity level.

The purpose of this study was to test the abovementioned hypothesis and to investigate association between body iron levels and creativity. To investigate this issue, we determined if and how individual differences in creativity (measured by DT) were associated with iron, using hair mineral analysis. Creativity was determined using the S-A creativity test (Society_For_Creative_Minds, 1969). To reveal the nature of the association, we also investigated associations between iron levels and dopamine-related states and traits as well as physical activity levels.

A previous study (Priya and Geetha, 2011) reported that hair mineral analysis serves as a best bio-indicator of body mineral levels. Hair has been recognized as a potential repository of all the elements that enter the body, and hair mineral levels indicate the composition on the basis of minerals accumulated over a long period of time (Priya and Geetha, 2011). Correlations between the concentrations of basic elements in the body and hair have been previously shown (Chłopicka et al., 1998; Kedzierska, 2003). Hair mineral analysis has an advantage over other methods of investigating body mineral levels because hair mineral levels are not subjected to rapid fluctuations in mineral intake and have long-term stability (Ayodele and Bayero, 2009).

As described above, creative cognition has a number of crucial roles in cultural life, and levels of irons have been shown to be associated with neurocognitive functions. Thus, body iron levels and their associations with creativity are of public interest.

## Methods

### Subjects

Data from 332 healthy, right-handed individuals (244 men and 88 women; mean age = 20.79 ± 2.03 years) were used in this study as part of an ongoing project investigating associations among brain imaging, cognitive functions, aging, genetics, and daily habits (Takeuchi et al., 2010a, 2011a,b,d,e, 2012b, 2013, 2014; Taki et al., 2010, 2011). Hair samples were obtained from the large study samples (369 males and 305 females), but data from subjects who had undergone either hair dying, hair bleaching, or hair permanent within 6 months were excluded from the present study to improve data quality. Only the data from 332 subjects were analyzed in this study. All subjects were university, college, or postgraduate students or subjects who had graduated from these institutions within 1 year before the experiment and had normal vision. None had a history of neurological or psychiatric illness. A history of psychiatric illnesses and/or recent drug use was assessed using our laboratory's routine questionnaire in which each subject answered questions relating to their current or previous experiences of any of a list of illnesses and listed drugs that they had taken recently. Handedness was evaluated using the Edinburgh Handedness Inventory (Oldfield, 1971). Written informed consent was obtained from each subject in accordance with the Declaration of Helsinki (1991). This study was approved by the Ethics Committee of Tohoku University.

### Creativity assessment

The S-A creativity test (Society_For_Creative_Minds, 1969) was used to assess creativity. As described in our previous studies (Takeuchi et al., 2010a,b, 2011a,b, 2012a), a detailed discussion of the psychometric properties of this instrument and how it was developed is found in the technical manual of this test (Society_For_Creative_Minds, 1969). The test is used to evaluate creativity through DT (Society_For_Creative_Minds, 1969) and it involves three types of tasks. The first task requires subjects to generate unique ways of using typical objects. The second task requires subjects to imagine desirable functions in ordinary objects. The third task requires subjects to imagine the consequences of 'unimaginable things' happening. The S-A creativity test provides a total creativity score, which was used in this study, as well as scores for the following dimensions of the creative process: (1) Fluency, which is measured by the number of relevant responses to questions and is related to the ability to produce and consider many alternatives. Fluency scores are determined by the total number of questions answered after excluding inappropriate responses or responses that are difficult to understand. (2) Flexibility, which is the ability to produce responses from a wide perspective. Flexibility scores are determined by the sum of the (total) number of category types that responses are assigned based on a criteria table or an almost equivalent judgment. (3) Originality, which is the ability to produce ideas that differ from those of others. Originality scoring is based on the sum of idea categories that are weighted based on a criteria table or an almost equivalent judgment. (4) Elaboration, which is the ability to produce detailed ideas (Society_For_Creative_Minds, 1969). Elaboration scores are determined by the sum of responses that are weighted based on a criteria table or an almost equivalent judgment. These four dimensions correspond to the same concepts as those of the Torrance tests of creative thinking (TTCT; Torrance, 1966). Scoring of the tests was performed by the Tokyo Shinri Corporation. Please refer to our previous studies (Takeuchi et al., 2010a,b) for more extensive details, including those on the psychometric properties of this test, sample answers to the questionnaire, and the manner in which the tests were scored.

The primary analysis was limited to the total creativity score and did not include the score for each dimension because this score was highly correlated with the total creativity score as well as with each other (all correlations between the scores of any two dimensions had simple correlation coefficients of >0.56). This is consistent with another group of rather similar DT tests (Heausler and Thompson, 1988), TTCT (Torrance, 1966). Heausler and Thompson (1988) concluded that the correlations among the subscales in TTCT were so high that each subscale could not provide meaningfully different information. Treffinger (1985) warned that independent interpretations of TTCT subscores should be avoided. Consistent with this notion, a previous study (Chávez-Eakle et al., 2007) that investigated the association between regional cerebral flow (rCBF) and each dimension revealed that different creativity dimensions correlated with rCBF in similar regions. Thus, we believe that using only the total creativity score serves the purpose of this study.

### Other psychological outcome measures

Other psychological measures used to assess dopamine-related traits and states (refer to Introduction for details) were also analyzed, to see the effects on dopamine related traits and state. These include the following: [A] Vigor subscale of the shortened Japanese version (Yokoyama, 2005) of the Profile of Mood States (POMS) (McNair et al., 1992), which measures participants' motivation. In our study, it was used to measure each participant's mood on the day of the experiment (Takeuchi et al., 2011a); [B] Novelty seeking score on a Japanese version (Kijima et al., 1996) of the Temperament Character Inventory (Cloninger et al., 1993), which was used to measure novelty seeking; [C] Extraversion scale on a Japanese version of the NEO Five-Factor Inventory (NEO-FFI) (Costa and McCrae, 1992), which was used to measure extraversion.

Further, data on the subjects' average daily physical activity level of the past one month were collected using a self-report questionnaire. The questionnaire consists of multiple choice questions with the following four options: 1, little; 2, have exercised a little (approximately 1000 steps or approximately 400 m); 3, have exercised modestly (from approximately 1000 to 4000 steps or from approximately 400 to 1600 m); or 4, have exercised very much (more than approximately 4000 steps or more than 1600 m).

### Hair acquisition and hair mineral analysis

Scalp hair samples (approximately 4-cm length, 0.1-g weight) were collected from each subject, with the hair cut as close to the scalp as possible. The hair samples were sent to La Belle Vie research laboratory and analyzed by established methods (Yasuda et al., 2005b, 2008, 2009, 2011, 2012; Munakata et al., 2006), as described below.

Hair sample of 75 mg was weighed into 50 ml plastic tube, and washed twice with acetone and then with 0.01% Triton solution, in accordance with the procedures recommended by the Hair Analysis Standardization Board (Cranton et al., 1982). The washed hair sample was mixed with 10 ml 6.25% tetramethylammonium hydroxide (TMAH, Tama Chemical) and 50 μl 0.1% gold solution (SPEX Certi Prep.), and then dissolved at 75°C with shaking for 2 h. After cooling of the solution to room temperature, internal standard (Sc, Ga, and In) solution was added and, adjusting its volume gravimetrically, the obtained solution was used for mineral analysis. The mineral concentrations were measured with inductively coupled plasma mass spectrometry (ICP-MS; Agilent-7500ce) by the internal standard method (Yasuda et al., 2005a,b, 2007), and are expressed as ng/g hair (ppb). For quality control of the mineral analysis, the human hair certified reference material supplied from the National Institute for Environmental Studies of Japan (NIES CRM no. 13) (Yoshinaga et al., 1997) was used.

### Statistical analysis

The hair iron concentrations were log-normally distributed, and logarithms of mineral levels in the hair were analyzed for all measures used. For statistical analysis, iron were converted to logarithms for use in the analyses, as reported in previous studies (Yasuda et al., 2005b, 2008, 2009, 2011, 2012; Munakata et al., 2006). The relationships among psychological variables and mineral levels were investigated using multiple regression analysis and PASW statistical software (ver. 18 for Windows; SPSS Inc., Chicago, IL, USA).

We investigated the associations among hair iron levels, S-A creativity test scores, POMS Vigor subscale scores, Novelty Seeking scale scores, Extraversion scale scores, and physical activity levels. Each multiple regression analysis investigated the associations between two of these six variables after correcting for the effects of age, sex, self-reported height, and body mass index (BMI), which was calculated from the self-reported height and self-reported weight. As a result, we performed 15 multiple regression analyses.

In all analyses, results with a threshold of *P* < 0.05, corrected for false discovery rate (FDR) using the graphically sharpened method (Benjamini and Hochberg, 2000), were considered statistically significant. The correction for multiple comparisons using this method were applied to the results of abovementioned 15 multiple regression analyses. FDR is the error rate in the set of comparisons that are called significant, or, in other words, the proportion of comparisons which are wrongly called significant. In other words, among the multiple tested results, 5% of the results determined to be significant through this method, are not truly significant. In FDR testing, if there is truly no signal anywhere in the tested results, an FDR-controlling method has the same control as a family-wise error correction. FDR-based methods have been shown to be more powerful and sensitive than other available approaches to multiple statistical testing (See Benjamini and Hochberg, 1995 for a full discussion; Genovese et al., 2002).

### Mediation analysis

In the face of the results that showed hair iron levels significantly correlated with novelty seeking, extraversion, and physical activity levels (which all significantly correlated with creativity), but iron levels did not correlate with creativity, we examined the possibility that hair mineral levels were directly related to novelty seeking, extraversion, and physical activity levels and not to creativity and that the effects of hair mineral levels on creativity, if any, were mediated by novelty seeking, extraversion, and physical activity levels. The mediation analyses when the outcome variable (here, creativity) and the independent variable (here, iron mineral level) are not significantly associated with each other, are controversial, so the analyses may have an exploratory nature. As described previously (Erickson et al., 2010), mediation analyses can be performed by running a series of multiple regression analyses. If a relationship exists between an independent variable (A) and an outcome variable (B), a third variable might mediate the relationship between A and B if controlling for the variance attributable to the mediator variable reliably reduces the variance in B explained by A.

Mediation analyses were performed using the indirect macro designed for SPSS (Preacher and Hayes, 2008). This macro uses bootstrapped sampling to estimate the indirect mediation effects. We here tested how the Novelty Seeking, Extraversion, or physical activity levels mediated the relationship between creativity and hair iron levels by testing three models for the three possible mediating variables that showed a significant association with creativity. In this analysis, 1000 bootstrapped samples were drawn with replacement from the dataset to estimate a sampling distribution for the indirect mediation pathway (i.e., the pathway from hair iron levels to one of Novelty Seeking, Extraversion, or physical activity levels to creativity).

There were three mediation analyses, and in all of the analyses, the outcome variable was creativity (S-A creativity test score) and the independent variable was hair iron levels. Novelty Seeking, Extraversion, and physical activity levels were the mediating variables in each of the three respective mediation analyses. The mediation models were controlled for variance from age, sex, height, and BMI.

## Results

### Basic data

**Figure 1:**
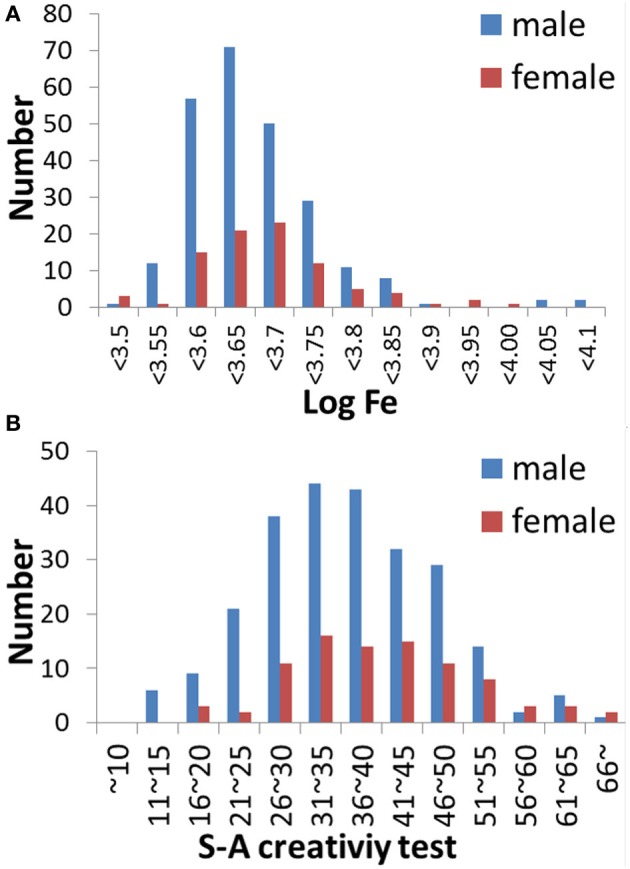
**Histograms of the variables. (A)** A histogram showing the logarithms of hair iron levels for male and female subjects. **(B)** A histogram showing S-A creativity test scores for male and female subjects.

Table 1 shows the average ± standard deviation (SD) values for age, scores for each psychological variable, and the logarithms of iron levels among the study participants. Figure 1 shows data on the distribution of the logarithms of iron levels and the distribution of S-A creativity test scores.

**Table 1:** **Psychological variables and n logarithms of iron among the study participants**.

**Measure**	**Mean**	**SD**
Age	20.79	2.03
S-A creativity	37.61	10.81
POMS-vigor	5.62	3.98
Novelty seeking	20.30	5.74
Extraversion	24.92	7.09
Physical activity level	2.45	1.10
Log-Iron (ppm)	3.66	0.09

### Correlations among variables

We investigated the association among hair iron levels; creativity, as measured by the DT test; vigor, as measured by POMS Vigor subscale score; Novelty Seeking scale score; Extraversion scale score; and physical activity levels after correcting for the effects of age, sex, height and BMI.

**Figure 2:**
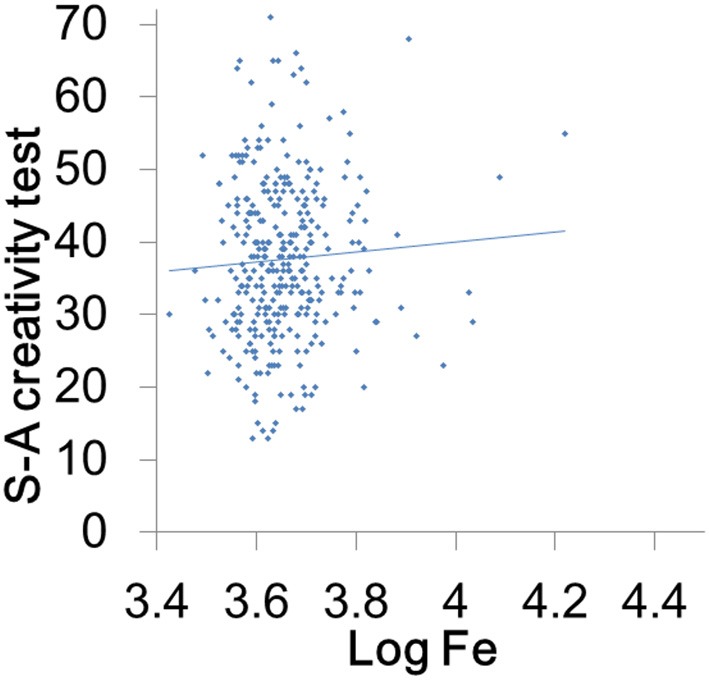
**Associations between hair iron levels and creativity.** A scatterplot comparing S-A creativity test score and the logarithms of hair iron levels, which shows a nonsignificant association with the trend line.

**Figure 3:**
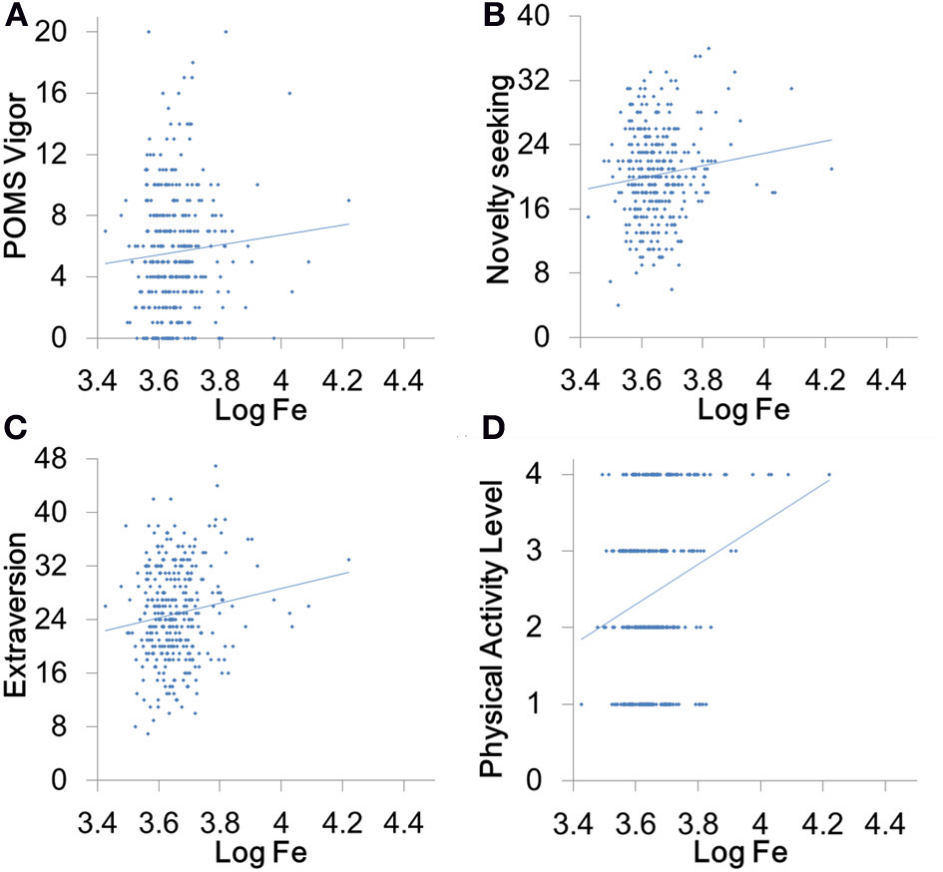
**Associations between hair iron levels and psychological variables related to creativity. (A)** A scatterplot comparing POMS Vigor subscale score and the logarithms of hair iron levels, which shows a nonsignificant association. **(B)** A scatterplot comparing Novelty Seeking scale score and the logarithms of hair iron levels, which shows significant positive associations. **(C)** A scatterplot comparing Extraversion scale score and the logarithms of hair iron levels, which shows significant positive associations. **(D)** A scatterplot comparing the physical activity level and the logarithms of hair iron levels, which shows significant positive associations.

Hair iron levels did not significantly correlate with creativity (Figure 2) and vigor (Figure 3A) but significantly and positively correlated with the scores of extraversion, novelty seeking, and physical activity levels (Figures 3B,C,D).

Further, congruent with the abovementioned previous studies, creativity was significantly and positively correlated with vigor, novelty seeking, extraversion, and physical activity levels.

Vigor was significantly and positively correlated with extraversion and the physical activity level. Novelty seeking was significantly and positively correlated with extraversion. Extraversion was significantly and positively correlated with the physical activity level.

For the statistical values of all of the results, please refer to Table 2.

**Table 2:** **Statistical values from the multiple regression analyses comparing mineral levels and cognitive functions**.

**Variables**	**Iron (log)**	**S-A creativity**	**POMS-vigor**	**Novelty seeking**	**Extraversion**	**Physical activity level**
Iron (log)	–					
S-A creativity	0.392 (0.176), 0.857, 0.047	–				
POMS-vigor	0.194 (0.102), 1.300, 0.072	0.005 (0.005)^*^, 2.802, 0.154	–			
Novelty seeking	0.039 (0.025)^*^, 2.073, 0.112	0.006 (0.005)^*^, 2.742, 0.151	0.355 (0.172), 0.927, 0.052	–		
Extraversion	0.020 (0.014)^*^, 2.337, 0.126	3.46^*^10^−7^ (7.27^*^10^−7^)^*^, 5.204, 0.279	3.78^*^10^−8^ (1.19^*^10^−7^)^*^, 5.635, 0.301	2.54^*^10^−8^ (1.19^*^10^−7^)^*^, 5.71, 0.301	–	
Physical activity level	6.68^*^10^−5^ (8.42^*^10^−5^)^*^, 4.040, 0.219	3.02^*^10^−5^ (4.76^*^10^−5^)^*^, 4.232, 0.226	0.055 (0.032)^*^, 1.926, 0.106	0.536 (0.225), 0.620, 0.034	0.002 (0.002)^*^, 3.052, 0.164	–

Values are as follows: uncorrected P-values (FDR-adjusted P-values), t-value, standardized partial regression coefficient (β).

* P < 0.05, corrected for FDR.

### Mediation analysis

Because of the abovementioned results, we examined the possibility that hair mineral levels were directly related to novelty seeking, extraversion, and physical activity levels and not to creativity and that the effects of hair mineral levels on creativity, if any, were mediated by novelty seeking, extraversion, and physical activity levels.

The results of mediation analyses showed that extraversion (*Z* = 2.302, *p* = 0.021) and physical activity levels (*Z* = 3.128, *p* = 0.002) significantly mediated the relationship between hair mineral levels and creativity. However, novelty seeking did not significantly mediate the relationship between hair mineral levels and creativity (*Z* = 1.484, *p* = 0.138).

The interpretations of causality in a mediation analysis are only valid as long as the assumptions of the direction of the effects between the variables are valid and all other potentially confounding variables are accounted for (Judd and Kenny, 1981; Baron and Kenny, 1986; Mackinnon et al., 1995; Erickson et al., 2010). In this study, the associations between hair iron levels and creativity were not significant; thus, the present results of the mediation analyses suggested that if an association between hair iron levels and creativity existed and if the assumed causality or direction of the effects were correct, then the association between hair iron levels and creativity was mediated by extraversion and physical activity levels.

## Discussion

To the best of our knowledge, this is the first study to investigate the associations between hair iron levels and creativity, and with dopamine-related traits, states, and behavioral variables. Partly congruent with our hypothesized mechanisms, higher iron levels did show a significant or near-significant tendency of associations with dopamine-related traits (novelty seeking and extraversion), which are robustly associated with creativity. These results may be congruent with the notion that iron plays a key role in the dopaminergic system. Further, higher iron levels also showed a significant positive correlation with physical activity levels, which were also robustly correlated with creativity. However, in contrast to our hypothesis, hair iron levels did not show any signs of an association with creativity. Future interventional or longitudinal studies are warranted to ascertain whether there are any causal effects between these associations.

High iron levels were associated with dopamine-related traits. Higher dopamine levels were significantly and positively correlated with the subjects' novelty seeking and extraversion. As previous studies have shown (King et al., 1996; Chavez-Eakle et al., 2006; Prabhu et al., 2008), these measures, as well as POMS Vigor subscale score, were significantly correlated with creativity. Considering the critical roles of dopamine functions in these traits (Ashby and Isen, 1999; Depue and Collins, 1999; Suhara et al., 2001; Schinka et al., 2002; Kaasinen et al., 2004; Tomer and Aharon-Peretz, 2004; Kaplan and Oudeyer, 2007; Bódi et al., 2009), these results appear to support the notion that iron levels in the body are essential for dopamine functioning (McCann and Ames, 2007). They may also support findings of significantly lower hair iron levels among individuals with neurological diseases associated with dopamine deficiency (Forte et al., 2005). However, as suggested previously, a simple lack of iron and oxygen in the brain due to lower body iron levels might cause the association between body iron levels and general brain functions including dopamine functioning. We suspected that this was unlikely to explain the associations observed, because the supplemental analyses showed that there were no associations between hair iron levels and the measure of general intelligence (Raven's Advanced Progressive Matrix test; see Takeuchi et al., 2010b for details of this test and how the data is gathered) in the present study when the association was tested using multiple regression analyses, with age, sex, height, and BMI as additional covariates (*P* > 0.1). However, iron levels were not significantly correlated with POMS Vigor subscale scores. This may have been due to the lack of statistical power, considering the trend line of the positive association between these two variables.

However, the present studies were not brain chemistry imaging studies. In addition, despite the present associations between dopamine-related traits and body iron levels and the animal studies of the association between body iron levels and the affinity and density of dopamine D2 receptors (Yehuda and Youdim, 1989; Beard et al., 1993; Youdim and Yehuda, 2000), it is not certain whether the variations in body iron levels among normal human samples are related to the states of the dopaminergic system and if those states are related to dopamine-related higher-order cognitive traits. To solve these issues definitively, positron emission tomography studies involving dopamine receptors (Bäckman et al., 2011) are warranted.

Hair iron levels were also significantly and positively correlated with the physical activity level, which was robustly and positively correlated with creativity. A number of mechanisms can underlie these associations. One possibility is that higher iron intake levels lead to higher hemoglobin and red blood cell levels, which possibly enables subjects to engage in more frequent physical activity (Hata, 1982) and sports without problems. It is possible that increased dopamine levels in the brain resulting from heightened iron levels, as suggested above, may facilitate increased physical activity levels. These are speculations, and we do not have available evidence to support them. Future studies are needed to investigate the possible mechanisms.

However, the iron level was not significantly correlated with creativity in this study. The reasons for this finding remain unclear, and we can only speculate weakly here and avoid going into the deep discussions about the mechanisms that underlie this finding. One possible reason could be that there are only indirect associations between creativity and iron levels and that these are mediated by other variables (novelty seeking, extraversion, vigor, and physical activity level), thus the association between creativity and iron was weak. Partly congruent with this notion, the exploratory mediation analyses revealed that an extraversion tendency and physical activity levels significantly mediated the association between hair iron levels and creativity, although the mediation effect of the novelty seeking tendency did not reach significance.

The present significant associations between iron levels and behavioral and psychological variables extended previous relevant findings reported in infants to adults using a large sample. As described in the Introduction, previous studies of infants with anemia, correlation studies of body iron levels, and intervention studies of iron supplementation have shown that lower body iron or hemoglobin levels are associated with higher levels of negative effects, lower levels of attention to people and objects, and activity levels (Lozoff et al., 1996, 1998, 2003; Wachs et al., 2005). The present findings of the positive associations between novelty seeking, extraversion, and physical activity levels were congruent with these previous results, considering the similarity of extraversion and novelty seeking with the measures reported in the previous study. Thus, it can be said that the present findings extended the previous findings to young adult samples using a larger sample size and suggested that these associations of temperament, personality, body activity levels, and body iron levels are not limited to infants without life experiences.

There are a few limitations to this study. One is that similar to majority of studies using hair mineral analysis, the present study was a cross-sectional study. Thus, despite the strength of the hair mineral analysis and the large study sample, any implications regarding causal effects cannot be viewed as definitive. To solve this problem, intervention studies of iron supplementation are warranted for determining whether iron supplementation can increase dopamine-related traits and physical activity levels. Through these studies, it can be determined whether iron intake can facilitate dopamine-related traits and body activity levels, both of which are essential parts of our social and physical everyday life. In addition, despite the importance of iron in the dopaminergic system, evidence is available that suggests iron accumulation in the brain helps the progression of neurological diseases (Zecca et al., 2004), and whether any detrimental effects of higher iron levels in the body of older subjects are observed, may have to be investigated in future studies. Finally, in this study, the study population was unbalanced toward males due to the low availability of hair that fulfilled these conditions of the study in females, and we did not and could not investigate gender-specific relationships between hair iron levels and psychological variables. The measures used in this study, such as creativity, were measured by DT tests that show gender differences. It is therefore possible that the relationship between iron levels and psychological variables may differ between females and males. Future studies are needed to investigate this issue.

Creative cognition and dopamine-related traits, states, and physical activity levels, which are related to creativity, are important aspects of our cultural and everyday life. Our findings showed that hair iron levels did not significantly and directly correlate with creativity but instead positively correlated with novelty seeking, extraversion, and physical activity levels. Our findings may imply the importance of iron intake, even in normal samples, for the facilitation of these traits and activity. Future longitudinal studies are warranted to confirm these notions.

### Conflict of interest statement

The authors declare that the research was conducted in the absence of any commercial or financial relationships that could be construed as a potential conflict of interest.
